# Proceedings of the second biennial Cleveland Neural Engineering Workshop 2013

**DOI:** 10.1186/s42234-018-0016-5

**Published:** 2018-12-05

**Authors:** Kim Anderson, Abidemi Ajiboye, Timothy Denison, Jennifer French, Kenneth Gustafson, Kevin Kilgore, Naomi Kleitman, Audrey Kusiak, Brian Litt, Megan Moynahan, Eric Perreault, Douglas Weber, Justin Williams, Dustin Tyler

**Affiliations:** 1 Dept of Physical Medicine and Rehabilitation, Case Western Reserve Univ, Cleveland, OH USA; 2 Cleveland VA Medical Center, Cleveland, OH USA; 3 Dept of Biomedical Engineering Case Western Reserve Univ, Cleveland, OH USA; 4 Nuffeild Dept of Clinical Neurosciences Oxford University, Oxford, UK; 5 Neurotech Reports, San Francisco, CA USA; 6 Dept of Orthopaedics MetroHealth Medical Center, Cleveland, OH USA; 7 Craig H Neilsen Foundation, Encino, CA USA; 8 Rehabilitation Research and Development Department of Veterans Affairs, Washington, DC, USA; 9 Dept of Neurology and Dept of Bioengineering Univ of Pennsylvania, Philadelphia, PA USA; 10 Institute for Functional Recovery, Cleveland, OH USA; 11 Dept of Biomedical Engineering Northwesten University, Chicago, IL USA; 12 Dept of Bio Engineering University of Pittsburgh, Pittsburgh, PA USA; 13 Dept of Biomedical Engineering Univ of Wisconsin-Madison, Madison, WI USA

**Keywords:** Neural, Engineering, Strategy, Infrastructure, Advocacy, Rehabilitation, Nervous system

## Abstract

The Cleveland Neural Engineering Workshop (NEW) is a biennial meeting started in 2011 as an “unconference” to bring together leaders in the neural engineering and related fields. Since the first iteration of the meeting, NEW has evolved from “just getting together” to a more important purpose of creating, reviewing, and promoting a uniform strategic roadmap for the field. The purpose of this short report, as well as the companion 2015 and 2017 reports, is to provide a historical record of this meeting and the evolution of the roadmap. These reports more importantly establish a baseline for the next meeting to be held in June, 2019. The second Neural Engineering Workshop (NEW) was held in June 2013. The two-day workshop was hosted by the Cleveland Advanced Platform for Technology National Veterans Affairs Center, the Functional Electrical Stimulation National Veterans Affairs Center, and the Case Western Reserve University in Cleveland, Ohio. Participants identified seven areas of future focus in the field of neural engineering: active communications with users, advocacy (regulatory), network building (clinical practice), case studies (clinical and technical), early industrial feedback, value chain resources, engagement, and advocacy (funding). This proceedings document summarizes the meeting outcome.

## Introduction

The goal was to bring together the neural engineering stakeholders with the specific purpose of developing a strategic plan, an infrastructure plan and best practices for the community. In June 2013 a select group of individuals were invited to participate in the Cleveland Neural Engineering Workshop (NEW). Individuals were selected based on their knowledge, contributions and advocacy to their respective fields. Action committees were comprised of 9–15 members. Each action committee was led by a provocateur(s) and included at least one executive committee member as a discussant (Table 1). Discussions from the members in attendance (Table 2) resulted in eight action items that the workshop identified as important to progress in neural engineering: active communications with users, advocacy (regulatory), network building (clinical practice), case studies (clinical and technical), early industrial feedback, value chain resources, engagement, and advocacy (funding). These items grew from initial discussion in the 2011 meeting (Table 3) and are summarized below.

**Table 1:** ClevelandNEW 2013 session summary. The table lists the eight planned sessions along with the last name(s) of the provocateur(s) and discussant assigned to lead those sessions

Session Title	Provocateur(s)	Discussant
Introduction	Tyler	Gustafson/Tyler
User/Consumer	French	Anderson
Regulatory/Reimbursement	Moynahan	Ajiboye
Clinical Practice	Litt	Weber
Technology/Innovation	Kilgore/Loeb	Perreault
Industry Translation	Denison	Williams
Funding	Kleitman/Kusiak	Gustafson
Summary	Tyler	Gustafson/Tyler

**Table 2:** List of ClevelandNEW 2013 workshop participants

Name	Institution (in 2013)	Name	Institution (in 2013)
Ajiboye, Bolu	Case Western Res Univ	Loeb, Gerald	Univ of S California
Anderson, Kimberly	Univ of Miami	Marasco, Paul	Louis Stokes Cle VA
Bensmaia, Sliman	Univ of Chicago	McIntyre, Cameron	Case Western Res Univ
Bourbeau, Dennis	Case Western Res Univ	Merrill, Dan	Ripple, LLC
Brill, Natalie	Case Western Res Univ	Miller, Jonathan	Case Western Res Univ
Brose, Steve	Louis Stokes Cle VA	Miller, Lee	Northwestern Univ
Capadona, Jeffrey	Case Western Res Univ	Mohseni, Pedram	Case Western Res Univ
Cullen, D. Kacy	Univ of Pennsylvania	Moynahan, Megan	Inst for Func Recovery
Denison, Timothy	Medtronic	Muthuswamy, Jit	Arizona State Univ
Durand, Dominique	Case Western Res Univ	Otto, Kevin	Purdue Univ
Fisher, Lee	Case Western Res Univ	Peckham, Hunter	Case Western Res Univ
French, Jennifer	Neurotech Network	Perreault, Eric	Northwestern Univ
Gaunt, Robert	Univ of Pittsburgh	Polacek, Laura	MetroHealth Med Cntr
Guillory, Shane	Ripple, LLC	Schiefer, Matt	CWRU & Cle VA
Gustafson, Kenneth	Case Western Res Univ	Sensinger, Jon	Rehab Inst of Chicago
Hess, Allison	CWRU & Cle VA	Solanki, Swarna	Case Western Res Univ
Johnson, Matthew	Univ of Minnesota	Triolo, Ronald	CWRU & Cle VA
Keith, Michael W.	MetroHealth Med Cntr	Tyler, Dustin	CWRU & Cle VA
Kilgore, Kevin	MetroHealth Med Cntr	Wagenaar, Joost	Univ of Pennsylvania
Kirsch, Bob	CWRU & Cle VA	Weber, Douglas	Univ of Pittsburgh
Kleitman, Naomi	Craig H. Neilsen Found	Williams, Justin	Univ of Wisconsin
Kusiak, Audrey	Dept of Veterans Affairs	Williams, Matt	Louis Stokes Cle VA
Litt, Brian	Univ of Pennsylvania	Zorman, Christian	Case Western Res Univ

**Table 3:** List of ClevelandNEW 2011 workshop participants

Name	Institution (in 2011)	Name	Institution (in 2011)
Ajiboye, Bolu	Cleveland FES Center	Lavik, Erin	Case Western Res Univ
Batista, Aaron	Univ of Pittsburgh	Lujan, Luis	Cleveland Clinic
Bikson, Marom	City Univ of New York	McIntyre, Cameron	Cleveland Clinic
Bourbeau, Dennis	Univ of Pittsburgh	Mohseni, Pedram	Case Western Res Univ
Bretl, Timothy	U. Illinois at U-C	Moran, Dan	Washington Univ
Brose, Steven	Cleveland FES Center	Murphey, Todd	Northwestern Univ
Bruns, Tim	Univ of Pittsburgh	Naqvi, Hassan	Cleveland Clinic
Butson, Christopher	Med Col of Wisconsin	Otto, Kevin	Purdue Univ
Capadona, Jeffrey	Case Western Res Univ	Peckham, P. Hunter	Cleveland FES Center
Carney, Paul	Univ of Florida	Perreault, Eric	Northwestern Univ
Chestek, Cynthia	Stanford University	Pinault, Gilles	Louis Stokes Clev VA
Cui, Xinyan	Univ of Pittsburgh	Putnam, David	Cornell Univ
Dorval, Chuck	Univ of Utah	Sachs, Nich	Northwestern Univ
Dukelow, Sean	Univ of Calgary	Schiefer, Matthew	Case Western Res Univ
Fridman, Gene	Johns Hopkins Univ	Shenoy, Krishna	Stanford Univ
Gaunt, Robert	Univ of Pittsburgh	Shoham, Shy	Technion
Gilbert, Ryan	Rensselaer Polytech Inst	Sloan, Andrew	University Hospitals
Gliha, Karen	n/a	Slutzky, Marc	Northwestern Univ
Gliha, Tom	n/a	Stegemann, Jan	Univ of Michigan
Gustafson, Kenneth	CWRU & Cle VAMC	Sutter, Maria	n/a
Hasenwinkel, Julie	Syracuse Univ	Taylor, Dawn	Clev Clinic & Cle VA
Helms-Tillery, Stephen	Arizona State Univ	Triolo, Ronald	CWRU & Cle VA
Hess, Allison	Case Western Res Univ	Tyler, Dustin	CWRU & Cle VA
Ho, Chester	Univ of Calgary	Ustin, Jeffrey	MetroHealth Med Cntr
Hoyen, Harry	MetroHealth Med Cntr	Wang, Wei	Univ of Pittsburgh
Jarosiewicz, Beata	Brown Univ	Weber, Doug	Univ of Pittsburgh
Kelly, Clay	Louis Stokes Cle VA	Wheeler, Don	n/a
Kirsch, Robert	Cleveland FES Center	Yu, Byron	Carnegie Mellon Univ
Kusiak, Audrey	Dept of Veterans Affairs	Zorman, Christian	Case Western Res Univ

## Active communications with users

Members of the workshop voiced concerns regarding communication between scientists and end-users. Scientists do not fully understand end-users’ needs (the input specifications), while end-users are not sufficiently aware of available technologies. There is insufficient communication between the end-user and the research enterprise. Therefore, improved bidirectional communication with the end-user is needed. Improved communication methods, consumer education programs, and common collective messaging might achieve this.

## Regulatory advocacy and reimbursement

The research community is insufficiently aware of Food and Drug Administration (FDA) regulations, upcoming changes to regulations, and the impact regulations have on research. Current regulations are predominately designed for commercial interests to achieve marketing approval. Testing requirements are suboptimal for early-phase, academic research. Moreover, increased requirements are becoming prohibitive to academic clinical research. As single voices, researchers have limited capability to change or affect the FDA. Therefore, stakeholders must join together to voice their concerns, as well as partner with larger interests in order to address the needs of the community.

## Network building for clinical practice

Members of the workshop recognized the complex challenges faced by clinicians when incorporating neural engineering into daily clinical practice. It was also recognized that inclusion of clinical colleagues in the development of neural technology would result in mutual benefits to scientists and clinicians. Building networks of clinicians interested in neural engineering may be an efficient and effective method to bridge the current communications gap. Clinician education is also an important step in building these networks and ultimately moving neural engineering into mainstream clinical practice. Therefore, there is a need for development of continuing education courses designed to train clinicians in neural engineering.

## Clinical and technical case studies

There is a paucity of accurate and objective sources regarding success or failure or neural engineering technology. This has led to dissemination of misinformation to stakeholders.

Similarly, there is a lack of a “best practices collection” for clinicians and researchers. This has led to individual reallocation of time and resources to solve challenges that may already have been addressed with success by others in the field. Therefore, there is a need for development of a clinical cases data and resource module that is user-friendly and scalable for the future.

## Early industrial feedback

The workshop members agreed, “We have a classical problem of building hammers and looking for nails.” The pathway from technology to implementation could be expedited if a feedback mechanism with industry was available early in the technology development process. Therefore, the community must develop best practices and create opportunities to engage in industrial feedback early on in the technology development life cycle.

## Value chain resources

Corporations employ models of technology assessment - a technology value chain. The value chains for different companies are different. Having insight into the value chains and pathways may help optimize the research and design process. Therefore, the goal is to develop a resource of this information available to the community.

## Engagement

There are significant challenges to securing funding in this space. One reason may be the lack of involvement by stakeholders. General funding development and the subsequent review process for awards would greatly benefit from improved engagement by researchers and leaders in the field. Currently, community leadership does not sufficiently engage in professional obligations such as review panels, advocacy in congress, and other national service-related activity. Therefore, the goal of this action committee is to engage in support and service.

## Advocacy for funding

Currently, there are assumptions and misinformation regarding funding, as well as lack of clarity by the research community, as to the appropriate funding mechanisms for their work. Ideally, a resource would be generated that would supply or connect the community to: funding resource road maps, information graphics, and other guides that are or may become available. This one-stop-shop of funding information should also be used to collect user feedback to assist in identifying funding mechanism appropriateness and utilization. In addition to appropriately allocating funding, it is of utmost importance that the research community provides information and justification for additional investments in new opportunities. Therefore, this action item will support consumer advocacy, veteran services organizations, Congress and appropriate lobbying organizations.

## Abbreviations

FDA: Food and Drug Administration
NEW: Neural Engineering Workshop

